# Recent Advances in NAMPT Inhibitors: A Novel Immunotherapic Strategy

**DOI:** 10.3389/fphar.2020.00656

**Published:** 2020-05-12

**Authors:** Ubaldina Galli, Giorgia Colombo, Cristina Travelli, Gian Cesare Tron, Armando A. Genazzani, Ambra A. Grolla

**Affiliations:** ^1^Department of Pharmaceutical Sciences, University of Piemonte Orientale, Novara, Italy; ^2^Department of Pharmaceutical Sciences, University of Pavia, Pavia, Italy

**Keywords:** nicotinamide adenine dinucleotide, nicotinamide phosphoribosyltransferase, cancer, inflammation, nicotinamide phosphoribosyltransferase inhibitors

## Abstract

Nicotinamide adenine dinucleotide (NAD) is a cofactor of many enzymatic reactions as well as being a substrate for a number of NAD-consuming enzymes (e.g., PARPS, sirtuins, etc). NAD can be synthesized *de novo* starting from tryptophan, nicotinamide, nicotinic acid, or nicotinamide riboside from the diet. On the other hand, the nicotinamide that is liberated by NAD-consuming enzymes can be salvaged to re-form NAD. In this former instance, nicotinamide phosphoribosyltransferase (NAMPT) is the bottleneck enzyme. In the many cells in which the salvage pathway is predominant, NAMPT, therefore, represents an important controller of intracellular NAD concentrations, and as a consequence of energy metabolism. It is, therefore, not surprising that NAMPT is over expressed by tumoral cells, which take advantage from this to sustain growth rate and tumor progression. This has led to the initiation of numerous medicinal chemistry programs to develop NAMPT inhibitors in the context of oncology. More recently, however, it has been shown that NAMPT inhibitors do not solely target the tumor but also have an effect on the immune system. To add complexity, this enzyme can also be secreted by cells, and in the extracellular space it acts as a cytokine mainly through the activation of Toll like Receptor 4 (TLR4), although it has not been clarified yet if this is the only receptor responsible for its actions. While specific small molecules have been developed only against the intracellular form of NAMPT, growing evidences sustain the possibility to target the extracellular form. In this contribution, the most recent evidences on the medicinal chemistry of NAMPT will be reviewed, together with the key elements that sustain the hypothesis of NAMPT targeting and the drawbacks so far encountered.

## Introduction

Among the hallmarks of cancer (Hanahan and Weinberg, 2011), re-programming of energy metabolism in a context of higher demand compared to healthy cells (Tennant et al., 2010) is possibly the one that has received the least attention. This is somewhat surprising given that the first data showing that tumoral cells undergo an important metabolic re-programming, switching from oxidative phosphorylation to aerobic glycolysis, were reported by the Nobel Prize Winner Otto Warburg over 80 years ago (Pavlova and Thompson, 2016).

Nicotinamide adenine dinucleotide (NAD) is an indispensable electron carrier in cellular energetics in key pathways, including glycolysis, the tricarboxylic acid (TCA) cycle, and oxidative phosphorylation. The higher demand of ATP of cancer cells, together with their metabolic reprogramming, undisputedly leads to a higher demand of NAD(P). This is further accentuated given that a number of key enzymes, over-expressed or over-activated in cancer, also consume NAD. For example, (i) mono- and poly-ADP ribosyltransferases (including ARTs and PARPs) transfer the ADP ribose moiety to acceptor proteins (Grimaldi and Corda, 2019), (ii) sirtuins catalyze the NAD^+^-dependent deacetylation of metabolic enzymes and transcription factors, thus controlling metabolism and gene transcription (Kosciuk et al., 2019); and (iii) CD38 uses NAD to generate a number of second messengers, including ADP ribose (ADPR), cyclic ADP ribose (cADPR), and nicotinic acid adenine dinucleotide phosphate (NAADP) (Deaglio et al., 2001). It has been calculated that cellular NAD turnover in cancer cells has a half-life of approximately 1 h (Rechsteiner et al., 1976).

Cells, and cancer cells in particular, therefore constantly require to replenish the NAD pool and a number of pathways that play this role exist. Mammalian cells can form NAD *de novo* from dietary precursors: tryptophan, nicotinic acid, nicotinamide or nicotinamide riboside (the biochemistry of NAD synthesis has been reviewed elsewhere; (Chiarugi et al., 2012)). Yet, in many cells, the liberated nicotinamide from NAD-utilizing enzymes can be re-used in a pathway known as the salvage pathway, and this becomes the predominant manner to maintain NAD levels in many cells. Briefly, nicotinamide phosphoribosyl transferase (NAMPT) catalyzes the synthesis of nicotinamide mononucleotide (NMN) from nicotinamide (NAM) and PRPP (in the presence of ATP). NMN is then converted to NAD by nicotinamide mononucleotide adenylyltransferase (NMNAT). Interestingly, the pathway from nicotinic acid is similar, with nicotinic acid phosphoribosyl transferase (NAPRT) substituting NAMPT.

Given the high turnover of NAD in cancer cells and the fact that NAMPT is the rate-limiting enzyme in the salvage pathway, inhibitors of this enzyme were first reported as possible anticancer agents by Hasmann et al. in 2003, who presented the first specific nanomolar inhibitor of this enzyme, FK866 (also known as APO866; (Hasmann and Schemainda, 2003). At the time, the rationale was mainly supported by the over-expression of NAMPT in cancer cells (a finding which has been reported in numerous cancer types, as reviewed in (Gallí et al., 2010; Galli et al., 2013; Sampath et al., 2015). (Heske et al., 2017; Audrito et al., 2018; Audrito et al., 2019; Lucena-Cacace et al., 2019; Zhu et al., 2019). This has led to a first wave of molecules that entered clinical trials for cancer, with no molecule reported to have progressed to later stages (www.clinicaltrials.gov; **Table 1**).

**Table 1:** Clinical Trial Data of NAMPT Inhibitors.

Drug	Ph	N°	Treatments	Condition or Disease	Outcomes	Results	DLTs^*^	State	ID
APO866	I/II	10	IV infusion at 0.126 mg/m^2^/h for 4 consecutive days (1 cycle)	B-cell chronic lymphocytic leukaemia	Safety and tolerability	Stable disease in most patients	Thrombocytopenia	Completed	NCT00435084 Holen et al., 2008
APO866	II	25	0.126 mg/m²/h IV every 4 weeks for 4 consecutive days (3 cycles)	Melanoma	Determine the tumor response rate	Stable disease in most patients	Thrombocytopenia	Completed	NCT00432107 Holen et al., 2008
APO866	II	25	0.126 mg/m²/h IV every 4 weeks for 4 consecutive days (3 cycles)	Cutaneous T-cell lymphoma	Safety and tolerability; tumor response	Stable disease in most patients	Lymphocytopenia; thrombocytopenia	Completed	NCT00431912 Goldinger et al., 2016
CHS-828 (GMX1778)	I	<50	Range of concentration, 20–500 mg once every 3 weeks PO	Solid tumors	Pharmacokinetics study	Stable disease in most patients or no results posted	Thrombosis; vomiting; diarrhea; thrombocytopenia; leucopenia	Withdrawn	NCT00003979 Hovstadius et al., 2002; Ravaud et al., 2005; von Heideman et al., 2010
GMX1777	I	19	60–200 mg/m^2^ 24-hour IV infusion once every 3 weeks	Solid tumors and lymphomas	Determine the recommended phase II dose	No results posted	GI hemorrhage; thrombocytopenia; tash	Withdrawn (due to financial constraints)	NCT00457574 Pishvaian et al., 2008
GMX1777	I/II	1 actual	Combination with temozolomide	Metastatic melanoma	Determine the recommended Phase II dose	No results posted	No results posted	Terminated (due to financial constraints)	NCT00724841
KPT-9274 (ATG-019)	I	175	Oral KPT-9274 three times a week every other day; 500 mg niacin ER co-administered with each dose of oral KPT-9274 three times a week every other day	Solid malignancies or non-Hodgkin's lymphoma (NHL)	Determine the Maximum tolerated dose (MTD) and DLTs	No results posted	No results posted	Recruiting	NCT02702492
KPT-9274 (ATG-019)	I	70	Alone: A starting does of 30 mg With niacin: A starting dose of 60 mg ATG-019 and 500 mg niacin ER	Solid tumors, non-Hodgkin's lymphoma	Determine the maximum tolerated dose (MTD) and DLTs	No results posted	No results posted	Not yet recruiting	NCT04281420
OT-82	I	50	The starting OT-82 dose level will be 16.5 mg/m^2^ given orally as an oral suspension once daily	Relapsed or refractory lymphoma	Determine the DLTs and overall response rate	No results posted	No results posted	Recruiting	NCT03921879

*DLTs, dose-limiting toxicities; Ph, phase; N°, number of patients; ID, clinical trial identifier.

FK866, CHS-828 (or GMX-1778), and its pro-drug (GMX-1777) were all tested in advanced hematological or solid malignancies, but the lack of significant anti-tumor benefits and the side effects observed dampened the enthusiasm in the field. Yet, this experience and the evidences gathered on NAMPT since have provided new cues onto which develop new drug programs in the field. In the present review, the most recent advances in the medicinal chemistry of NAMPT targeting agents will be presented.

**Figure 1:**
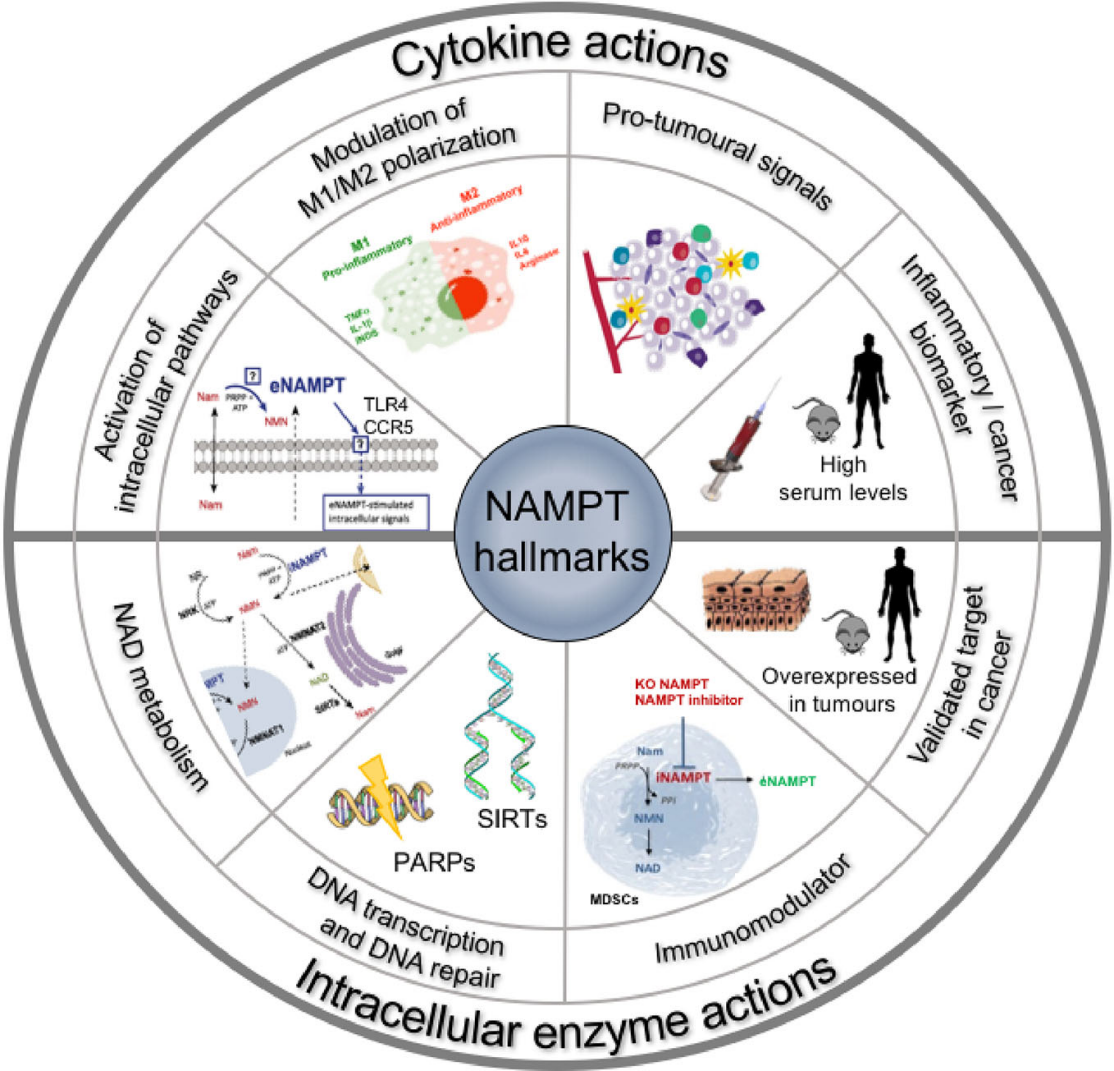
Hallmarks of NAMPT: the roles of the extracellular and intracellular forms of NAMPT.

For space limitations, we refer to recent reviews (Montecucco et al., 2013a; Sun et al., 2013; Garten et al., 2015) for most of the background on NAMPT (**Figure 1**). Yet, we feel important to briefly outline four aspects of these inhibitors that are often overlooked in medicinal chemistry programs: (i) their potential toxicity; (ii) the fact that the target protein is dual-faced, with an intracellular form and an extracellular form that may have different physiopathological roles; (iii) the fact that the tumoral cell might not be the sole target, or the most important target, of these inhibitors in cancer; (iv) the possible role of NAPRT in determining NAMPT inhibitor sensitivity or resistance.

### Toxicity of NAMPT Inhibitors

As mentioned above, toxicity of NAMPT inhibitors proved in clinical trials to be dose-limiting. Trombocytopenia has been the most significant dose-limiting toxicity in patients treated in clinical trials in phases I and II of solid tumors. These evidences were recapitulated in *in vivo* pre-clinical models and suggest that thrombocytopenia is on-target but occurs only at high doses and that other bone marrow-related toxicities, such as anemia and neutropenia, are also likely (Olesen et al., 2010; Tarrant et al., 2015).

Alongside hematological toxicities, it has been suggested in preclinical studies that retinal toxicity and cardiac toxicity may also be dose-limiting side effects, although this was not reported in the clinical trials (Misner et al., 2017; Cassar et al., 2018). While these have been described as on-target side effects, a recent article from our group somehow suggested that not all NAMPT inhibitors may be endowed with these risks, as we found inhibitors of NAMPT which lacked retinal and cardio-toxicity (Sun et al., 2013). This issue remains to be investigated further and may be due to the physiochemical properties of the compounds (e.g. lipophilicity). Lastly, the possibility that NAMPT inhibitors may induce hepatic steatosis has also been postulated in animal models (Wang et al., 2017), again with no clinical correlate yet.

In conclusion, therefore, the whole of the evidence suggests that toxicity of NAMPT inhibitors as single agents is most likely severe, similar to traditional chemotherapeutic drugs, and therefore regimens that mitigate this should be sought, including the possibility to boost the activity of NAPRT in healthy cells (see below).

### NAMPT Is a Dual-Faced Protein: Inside and Outside of Cells

The initial observations and rational to develop NAMPT inhibitors were based on the role of NAMPT as an intracellular enzyme. Yet, since then, it has been amply shown that NAMPT can be secreted by cells and may act as a cytokine-like protein. Whether NAMPT is free in serum or is trapped in microvesicles also requires to be firmly established (Yoshida et al., 2019), and most of the evidences correlating extracellular NAMPT and cancer have been reviewed in Grolla et al. (2016).

Serum NAMPT levels have been shown to be increased and often to correlate with cancer prognosis (reviewed in Galli et al., 2013; Shackelford et al., 2013). The literature strongly sustains a rationale to target also the extracellular protein in cancer and inflammatory diseases. It is likely that this role does not involve the enzymatic activity of the protein, although this is still a controversial issue (Revollo et al., 2007; Hara et al., 2011). Most of these evidences have been reviewed in Grolla et al. (2016) and are summarized in **Figure 1**.

The exact interplay between the intracellular and extracellular forms has still to be fully clarified, and therefore, it is almost impossible to ascertain whether the effect of the inhibitors so far developed and their potential toxicity can be in part ascribed to the binding to the extracellular form. Furthermore, both appear to be plausible targets in oncology, and approaches can be devised to target only the extracellular form. Novel molecules should, therefore, be designed and characterized for their effect on the intracellular enzymatic activity and/or on the effects of the extracellular form.

### Novel Mechanism of Actions of NAMPT Inhibitors

The most important advance in the field in recent years has been the determination that the intracellular and the extracellular forms of NAMPT are linked to inflammation, and may be directly connected to an effect on the tumor microenvironment. Briefly, NAMPT contributes to myeloid biology, governing monocyte/macrophage differentiation, polarization, and migration (Travelli et al., 2018). For example, the importance of NAMPT in M1 macrophages is supported by the report that FK866 drives the down-regulation of TNFα and IL-6 expressions. In support of this, Al-Shabany et al. demonstrated that NAD levels are increased in M1-like THP-1 cells and that LPS increases the NAD pool in cells and the relative expression of NAMPT, IDO (indoleamine 2,3-dioxygenase) and CD38, two other key enzymes that govern NAD signaling (Al-Shabany et al., 2016). Interestingly, this is paralleled by the up-regulation of PPARγ and CD163, markers of M2 macrophages (Audrito et al., 2015). Not only macrophages but also T lymphocytes undergo massive NAD depletion upon NAMPT inhibition and, as a consequence, impaired proliferation, reduced IFNγ and TNFα production, and eventually autophagic cell demise (Bruzzone et al., 2009). This is supported by the demonstration of how intracellular NAD promotes TNF synthesis by activated immune cells through sirtuin 6 (Van Gool et al., 2009).

A role of NAMPT in inflammation is also supported by the number of reports that have shown an effect of NAMPT inhibitors in inflammatory models (Montecucco et al., 2013b; Camp et al., 2015; Chen et al., 2017; Travelli et al., 2017; Gerner et al., 2018; Franco-Trepat et al., 2019), but is also directly supported by evidences in cancer biology. Indeed, it has been recently reported that myeloid-specific ablation of NAMPT or NAMPT inhibitors themselves prevent myeloid-derived suppressor cell (MDSC) mobilization, reactivated specific antitumor immunity, and enhance the antitumor activity of immune checkpoint inhibitors (Travelli et al., 2019b). Travelli et al. have also demonstrated that in cancer a multistep metabolic process is guided by increased levels of NAMPT, which result in the inactivation of the CXCR4-dependent retention axis and mobilization of suppressor myeloid populations toward tumor immune suppression. All this leads to the observation that the effect of NAMPT inhibitors on tumor growth and metastasis formation is largely independent of an effect on the tumors but is mediated by an effect on the microenvironment. For example, in tumors that are rendered insensitive to NAMPT inhibitors (by mutating the enzyme in such a way that it is unable to bind to these compounds), there is still an important effect of the inhibitors themselves in tumor-bearing mice (Travelli et al., 2019b). In support of this, NAMPT inhibitors enhance the anti-tumor efficacy of immune checkpoint inhibitors (i.e. antibody against PD-1; (Soncini et al., 2014; Travelli et al., 2019b). This finding modifies substantially the paradigm of the use of NAMPT inhibitors in cancer, as their use is no longer driven solely by the idea of strangling tumoral cells, but is also guided by the possibility of acting on the immune system. Given that this may occur at different concentrations or may require drugs with different physio-chemical properties, it brings new enthusiasm and fuel to the field.

The paradigm may change even further, though, as it is possible that NAMPT is necessary and may be targeted also for other processes. For example, over-expression of NAMPT has been associated to epithelial to mesenchymal transition (EMT) (Soncini et al., 2014). This is supported by a recent report that shows that FK866 is able to inhibit dose-dependently EMT in hepatocarcinoma (Zhang B. et al., 2018). Also, NAMPT has been found involved in tumor-associated angiogenesis. For example, Pylaeva et al. (2019) demonstrated that pro-angiogenic properties of neutrophils depend on the activation of NAMPT signaling pathway in these cells and inhibition of this pathway in tumor-associated neutrophils leads to their potent anti-angiogenic phenotype. Lastly, it has been recently shown that NAMPT is critical for the pro-angiogenic activity of tumor-associated neutrophils (TANs). TANs regulate many processes associated with tumor progression, and depending on the microenvironment, they can exhibit pro- or antitumor functions. The authors demonstrated, using transplantable tumor models, that NAMPT is essential for tumorigenic conversion of TANs and their pro-angiogenic switch and inhibition of NAMPT in TANs leads to their antitumor conversion (Pylaeva et al., 2019). All these data, taken together, therefore, show that NAMPT inhibitors may be considered dual agents that act on the tumor and on the microenvironment.

Lastly, NAMPT might be up-regulated as a resistance pathway operated by cancer cells, and therefore, NAMPT inhibitors could be used together with other oncological drugs to prolong their efficacy or after these drugs in subsequent lines of therapy. For example, Deaglio's group recently showed that extracellular NAMPT levels were elevated in 113 patients with BRAF-mutated metastatic melanoma compared to 50 with localized disease and to 38 healthy donors, showing a direct correlation with markers of tumor burden, such as LDH or aggressive disease (such as PD-L1). Importantly, NAMPT concentrations decreased in response to therapy with BRAF/MEK inhibitors, but increased again at progression (Audrito et al., 2018).

### NAPRT Is an Important Counterpart to NAMPT in NAD Metabolism

Potent NAPRT inhibitors have never been developed, and for a long time in the literature, the feeling that this protein might have had a marginal role was predominant. Recent evidences, instead, demonstrate that this is not the case.

**Figure 2:**
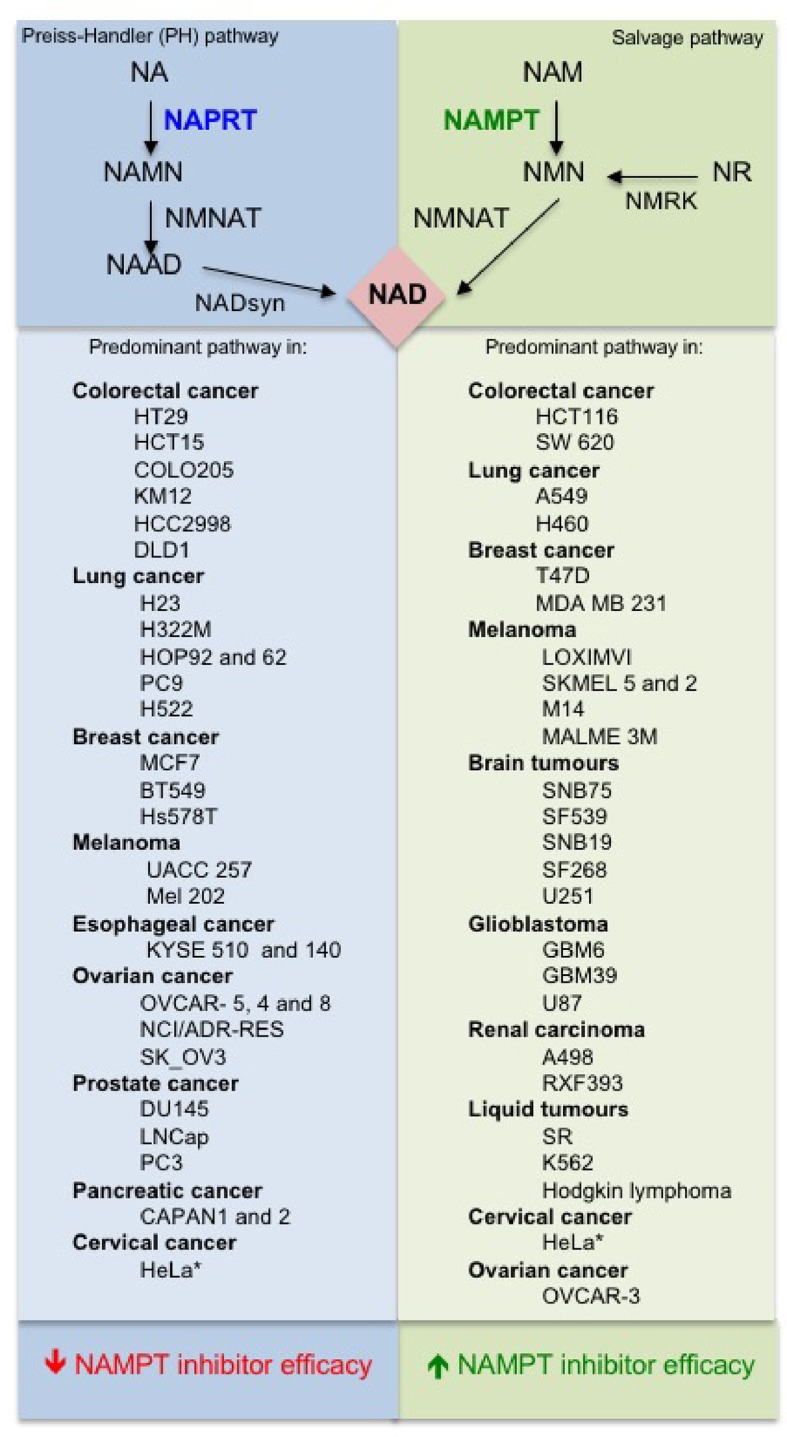
Predominant NAD pathways in different cancer cells (Sociali et al., 2016; Lee et al., 2018; Chowdhry et al., 2019).

The molecular mechanisms that dictate the choice of the NAD synthesis pathway (NAPRT and/or NAMPT) in normal or cancer cells is still not clear. A very recent and extensive work tackled this issue. Chowdhry et al. (2019) analyzed more than 7,000 tumors and 2,600 matched normal samples and found that if NAPRT is highly expressed in a normal tissue type, cancers that arise from that tissue will have a high frequency of NAPRT amplification and most likely will be dependent on NAPRT (Preiss-Handler pathway) for survival. By contrast, non–Preiss-Handler pathway amplified cancer cell lines depend exclusively on NAMPT and the salvage pathway (**Figure 2**). To note, cells in the nervous system express almost exclusively NAMPT (Duarte-Pereira et al., 2016) and, therefore, most of the brain tumors are predominantly based on salvage pathway (Chowdhry et al., 2019).

The above observations suggest that there is a strong need for NAPRT inhibitors to be developed and tested, but also that NAMPT inhibitors could be tailored to those tumors in which the Preiss-Handler pathway is irrelevant. Although the article by Chowdhry et al. suggests that dependence on the NAD pathway in cancer arises from tissue lineage-based gene amplification and epigenetic remodeling (Chowdhry et al., 2019), differences among tumors of identical origin are possible (Lee et al., 2018) and, therefore, screening of tumors is still essential. Furthermore, NAMPT-dependent and NAPRT-dependent tumors of the same origin may have different biological features. For example, it has been demonstrated that cell lines and tissues with EMT-associated gene expression signatures express low levels of nicotinic acid phosphoribosyltransferase (NAPRT), which makes these hypersensitive to NAMPT inhibition (Lee et al., 2018).

When the first inhibitors were developed, the role of NAPRT was possibly underestimated. Surprisingly, furthermore, a recent publication has also shown that NAPRT shares the same topological paradox (Managò et al., 2019). Just like NAMPT, extracellular NAPRT enhances monocyte differentiation into macrophages, and these effects are independent of NAD-biosynthetic activity (Managò et al., 2019).

All the elements above strongly suggest, therefore, that NAPRT expression should be considered when selecting malignancies that may be susceptible to NAMPT inhibitors. For example, NAPRT is amplified and overexpressed in a subset of common types of cancer, including ovarian cancer, where its expression correlates with a BRCAness gene expression signature.

The interplay between NAMPT inhibitors and NAPRT already has strong foundations (Buonvicino et al., 2018). For example, it has been shown that in cancer cells that lack NAPRT expression (Olesen et al., 2010; Duarte-Pereira et al., 2016) and in which NAD synthesis solely depends on NAMPT, NAMPT inhibitors are significantly more potent. In support of this ability of the two enzymes to vicariate for each other, it has been shown that silencing NAPRT (or inhibiting its activity with 2-hydroxynicotinic acid) sensitizes cells to NAMPT inhibitors (Piacente et al., 2017). Further support is brought by a patent that discloses chemical compounds that inhibit NAPRT activity (including some non-steroidal anti-inflammatory drugs) and sensitize cancer cells to NAMPT inhibitors (Krieg et al., 2013). From a different angle, it has been postulated that more aggressive therapies with NAMPT inhibitors could be envisaged against tumors that solely express NAMPT provided that a supplementation of the therapy with nicotinic acid is provided to boost NAD metabolism in healthy cells, while the tumor would be fully deprived of NAD. This would significantly widen the therapeutic index of NAMPT inhibitors. While this would appear plausible, retinal toxicity was not found diminished when animals were given a NAMPT inhibitor supplemented with nicotinic acid (Zabka et al., 2015). From a different angle, it has been recently described in a patent that chemical compounds that inhibit NAPRT activity (including some non-steroidal anti-inflammatory drugs) sensitize cancer cells to NAMPT inhibitors (Krieg et al., 2013). In this regard, Fons et al. (2019) demonstrated that mutant PPM1D, a protein phosphatase often found truncated in pediatric gliomas, drives hypermethylation of CpG islands throughout the genome and promotes epigenetic silencing of NAPRT, conferring NAMPT inhibitor sensitivity in glioma and revealing a promising approach for the targeting of PPM1D mutant tumors.

In this context, it should be noticed that quinolinate phosphoribosyltransferase (QPRT), which belongs to the phosphoribosyltransferase family and is involved in *de novo* NAD biosynthesis using quinolinic acid (QA) as a precursor in both prokaryotes and eukaryotes, has not been thoroughly investigated in the context of cancer and inflammation, but there are evidences that also this enzyme might play a role (Hinsch et al., 2009; Sahm et al., 2013; Ullmark et al., 2017; Haslinger et al., 2018).

In conclusion, therefore, the interplay between NAMPT and NAPRT must be considered when developing novel NAMPT inhibitors, when screening them for activity, when choosing the oncological setting in which to test them, and when attempting to improve their therapeutic index, thereby turning a threat into an opportunity. The possibility of developing non-selective inhibitors, targeting both NAPRT and NAMPT, considering the similarity of the two enzymes, could also be envisaged.

### Recent Advances in the Medicinal Chemistry of Intracellular NAMPT Inhibitors

**Figure 3:**
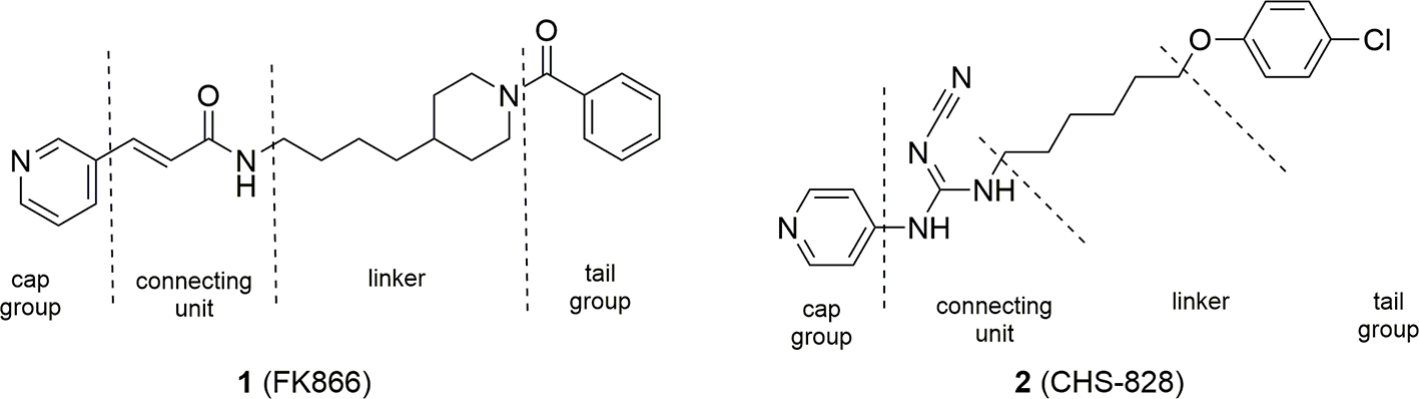
FK866 and CHS-828: the first potent NAMPT inhibitors discovered.

The seminal discovery that FK866 (**1**) was able to inhibit NAMPT with a one-digit nanomolar potency (Hasmann and Schemainda, 2003), and the disclosure of the crystal structure of FK866 in complex with the enzyme (Khan et al., 2006) initially boosted this area of research. Two years later, NAMPT was also identified as the principal biological target of the potent cytotoxic agent CHS-828 (**2**) (Olesen et al., 2008), which had already been described in the literature. Comparison between the two molecules brought to the identification of a common pharmacophoric structure for NAMPT inhibitors based on the topological nature of the binding site. Indeed, the binding site of the enzyme is a tunnel formed by the head to tail dimerization of two NAMPT units. A pyridine (or a pyridine like heterocyclic ring) as cap group mimics and binds at the same position of nicotinamide, the natural substrate of NAMPT. Then, a connecting unit, such as an amide, with the ability to accept hydrogen bonds, is bound to a hydrophobic linker characterized by an appropriate length and geometry able to protrude from the tunnel. Finally, a tail group which juts out over the solvent-exposed region complete the pharmacophoric model (**Figure 3**).

These four necessary pieces have been assembled like a lego-game by different research teams both in industry and academia giving a cornucopia of potent NAMPT inhibitors with the intent to identify molecules with better ADME properties and/or gaining a leading position in the field of intellectual properties. Structures emerging from this gold rush have been extensively reviewed until 2014. In this manuscript, we would like to review the novel inhibitors that have appeared in the literature or in patents from begin of 2015 until the end of 2019 and for the sake of clarity we have divided the inhibitors in three sub-groups: (i) molecules which follow the typical pharmacophoric model; (ii) molecules which contravene the classical pharmacophore model; (iii) dual hybrids where the NAMPT inhibitory activity is associated with another pharmacological action. We will not consider the antibody-drug conjugates (ADCs) which use NAMPT inhibitors as payloads, as this topic has been recently well reviewed (Neumann et al., 2018).

It is important to stress that most of the inhibitors so far described, even the most recent ones, have looked at tumor metabolism as the primary target (thereby overlooking all the other mechanisms), have not tackled the issue of toxicity and have not considered the interplay between NAPRT and NAMPT, thereby falling short of being game changing. Nonetheless, these molecules and the ones that will follow could be excellent tools to tackle these issues. Last, cross-resistance to a diverse set of NAMPT inhibitors has been already reported and demonstrated and therefore warrants for diverse inhibitors to be developed. Different mechanisms are responsible for resistance which include point mutation in the tunnel region, as well as increased expression of quinolate phosphoribosyltransferase an enzyme involved in the *de novo* NAD synthesis (Guo et al., 2017; Ogino et al., 2018).

#### **Molecules** W**hich** F**ollow the** Classic Pharmacophore Model

**Figure 4:**
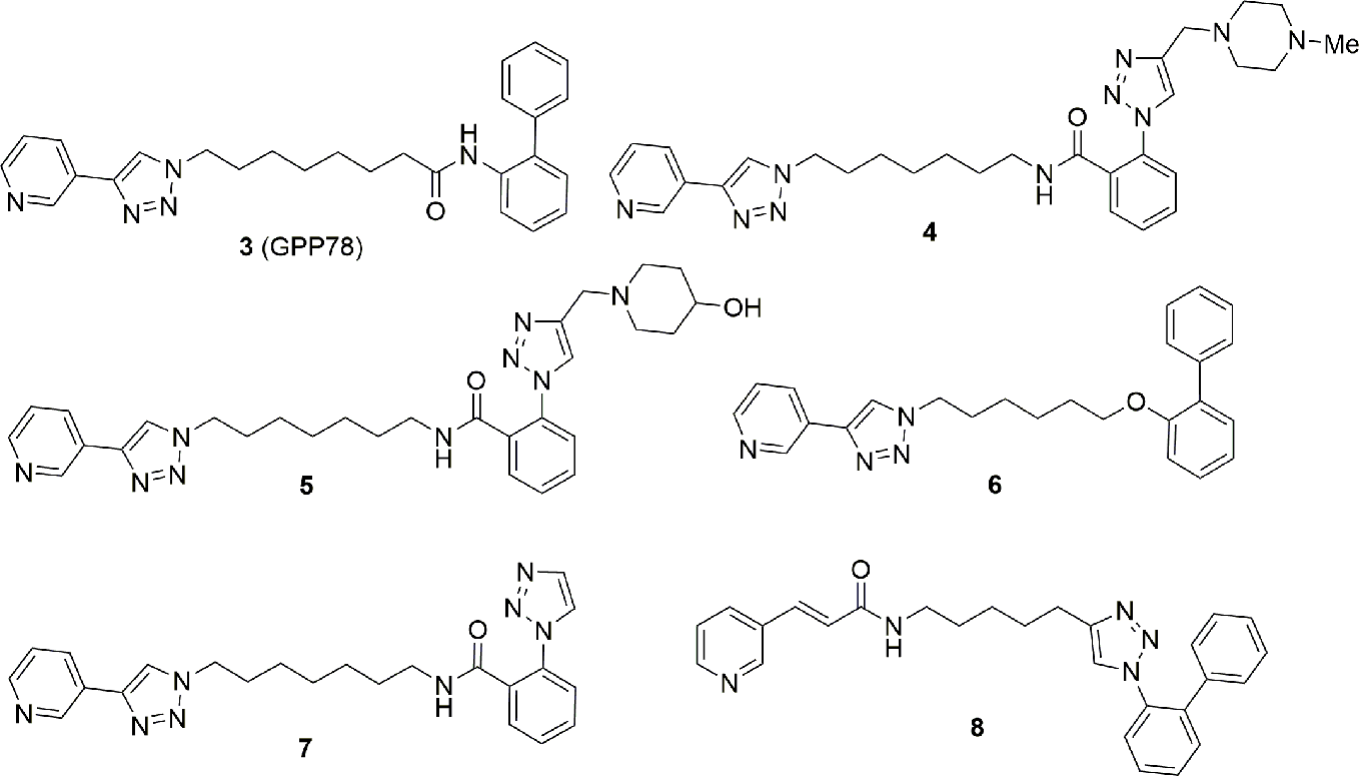
The most important triazole containing molecules as NAMPT inhibitors discovered to date.

After our discovery of the triazolylpyridine GPP78 (**3**) in 2010 using the click chemistry approach (Colombano et al., 2010), we decided to modify the solvent exposed region of FK866 both to improve the metabolic stability and to target the extracellular form of NAMPT. Metabolic stability was gained inserting non-hydrolysable functional groups in the tail region while selective eNAMPT targeting was tackled by adding a series of polar and ionizable polar groups in the tail region in order to prevent the molecules ability to cross cell membrane. The latter strategy brought to the identification of a series of molecules, namely, compounds **4** and **5**, which inhibit the enzyme NAMPT *in vitro*, lack cell cytotoxicity and are unable to cross the plasma membrane (**4**: IC_50_ NAMPT 13.6 ± 2.9 nM, EC_50_ SH-SY5Y > 1000 nM; **5**: IC_50_ NAMPT 41.8 ± 4.1 nM, EC_50_ SH-SY5Y > 1000 nM). Whether the enzymatic activity of the extracellular form plays any physiological or pathological role is, at present, controversial, and this tool might help those that wish to tackle this issue. On the other hand, **6** and **7** were identified as better NAMPT inhibitors with respect to the GPP78. In particular, **7**, possibly due to the high polarity, in our hands lacked retinal and cardiac toxicity, possibly suggesting that despite the fact that this is an on-target effect, it may be significantly mitigated. The same compounds were shown to be efficacious in reducing growth of triple negative mammary allograft carcinomas in mice (Travelli et al., 2017; Travelli et al., 2019a) (**Figure 4**).

With the intention to identify novel tail groups, we again capitalized on the click chemistry approach maintaining a substituted pyridine at the 3 position as cap group and synthesizing molecules with a 1,2,3-triazole ring in the tail position. Due to the high versatility of click chemistry, we were able to synthesize 720 new compounds which were screened for cytotoxicity and for cytotoxicity. This led to the description of a compound (EC_50_ of 20 nM for cytotoxicity on SH-SY5Y cells and an IC_50_ of 100 nM for enzyme inhibition) which bears a novel tail group **8** (Theeramunkong et al., 2015) (**Figure 4**).

**Figure 5:**
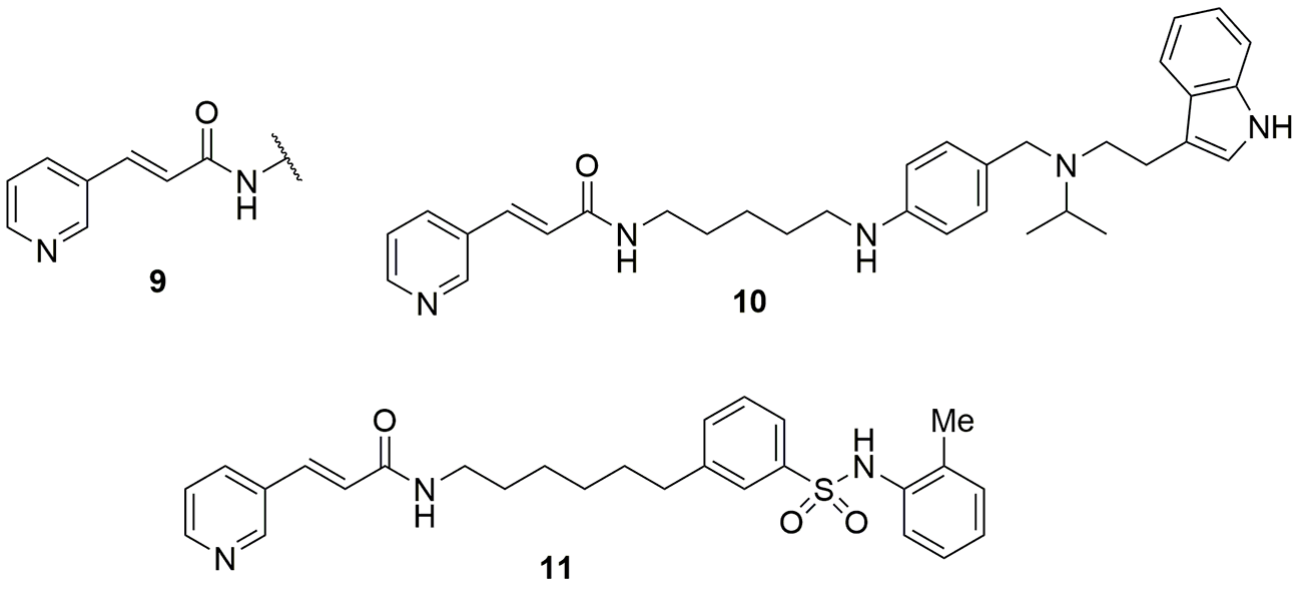
*trans* 3-(pyridin-3-yl)acrylamide cap group was pivotal for the identification of novel NAMPT inhibitors.

Other authors followed the same reasoning using the *trans* 3-(pyridin-3-yl)acrylamide portion **9** of FK866 as a template for the identification of new NAMPT inhibitors with the variation of the linker and tail group. In particular compound **10** demonstrated to be very potent, with acceptable *in vivo* pharmacokinetic properties, being efficient in tumor xenograft models (Bai et al., 2016). As mentioned above, the use of xenografts to characterize these molecules should nonetheless be discouraged, as it does not probe the contribution of the immune system in mediating the effect of NAMPT inhibitors. The same strategy was again applied by the same group which reported a different series of potent NAMPT inhibitors identifying biarylsulfanilamides moieties as tail groups. In particular, compound **11** was the most potent NAMPT inhibitor identified in this study with an IC_50_ of 5 nM, and with a potent antiproliferative activity (IC_50_s between 200 and 2 nM) against a number of cell lines (DU145, Hela, H1975, K562, MCF-7, and HUH7) (Zhang et al., 2019) (**Figure 5**).

**Figure 6:**
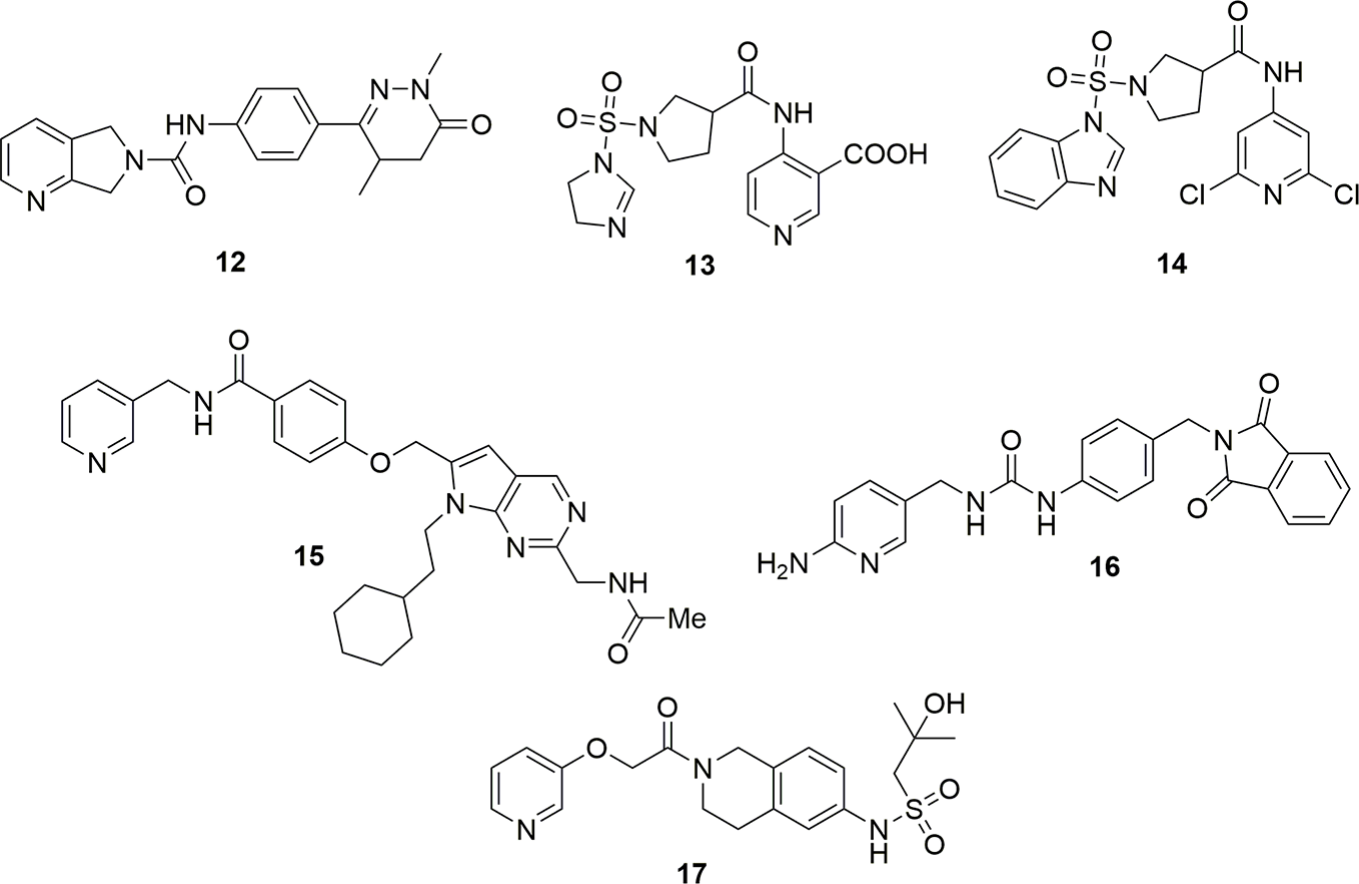
Structure of NAMPT inhibitors discussed in the text.

Molecules with a dihydropyrrole pyridine as cap group and dihydropyridazinones as tail group exemplified by molecule **12** have also been reported as potent NAMPT inhibitors (Giese et al., 2018) (**Figure 6**). Patent literature also shows that the cap group can be a nicotinic acid substituted at the 4 position (Tian, 2018a; Tian, 2018b). In the case of the molecule **13**, the IC_50_ on NAMPT inhibition was of around 3 nM. Molecules with a more canonical 2,6-dichloropyridine cap group have also been disclosed as potent NAMPT inhibitors (Tian, 2018c). Unfortunately, only the cytotoxic activity on a panel of cancer cell lines was reported for compound **14** (EC_50_ around 6 µg/mL on K562), and no inhibitory activity on the enzyme has been reported (**Figure 6**). The data on these compounds must, therefore, be considered with caution until it is proven that they inhibit NAMPT, as in our experience when the positions 2 and 6 of pyridine are substituted with bulky groups (e.g., chlorine and methyl), NAMPT inhibitory activity is abolished (UG and GCT personal communication). It is then possible to speculate that the cytotoxic activity for this compound might be due to the interaction with another pharmacological target.

Structural modifications aimed at simplifying the synthetically complex structure of compound **15** identified through a phenotypic screening (Estoppey et al., 2017) brought to the identification of compound **16** which, beyond being easily synthetizable, showed a remarkable activity (IC_50_ = 4.2 nM, EC_50_ (A2780) = 7 nM (Palacios et al., 2018) (**Figure 6**). To note that a 2-aminopyridine has been used as cap group in order to increase the basicity of the nitrogen atom of pyridine which should potentiate the hydrogen bond between Tyr18 and the nitrogen atom.

Eli-Lilly discovered a novel series of oral available NAMPT inhibitors which are exemplified by compound LSN3154567 (**17**) which has an IC_50_ of around 3 nM and a cytotoxic activity of about 10 nM after 24 h of treatment (Zhao et al., 2017) (**Figure 6**).

**Figure 7:**
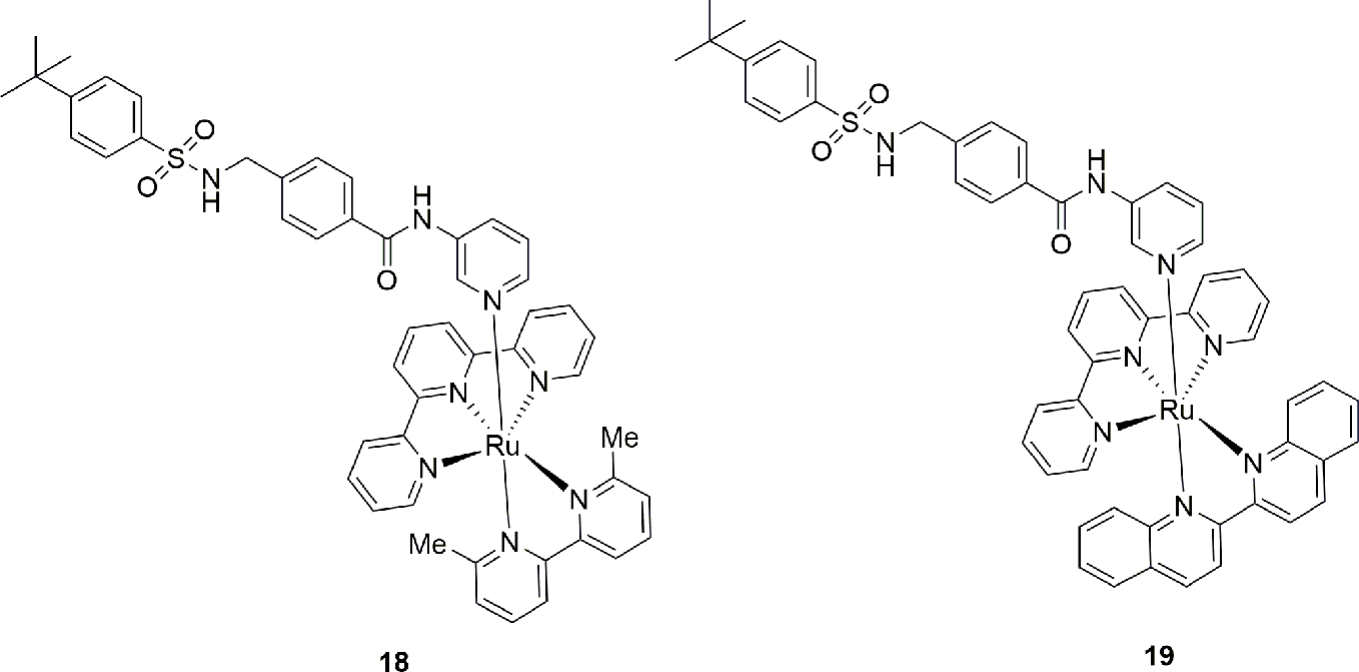
Photoactivable pro-drug NAMPT inhibitors.

A novel pro-drug strategy named photoactivated chemotherapy (PACT) was used with the intent to increase the water solubility of the NAMPT/GLUT1 inhibitor STF-31 and to reduce its side effects (Lameijer et al., 2017). In brief, two water soluble ruthenium complexes (**18** and **19**) were prepared using the pyridine nitrogen atom of the cap group as ligand. The main feature of these complexes, that are inactive *per se*, is that they can be photo dissociated when exposed to a low dose of red light. To note that the use of a red light instead of blue or green light allows for a deeper tissue penetration (0.5–1.0 cm) being also safer. Being oxygen not required for this photo dissociation, the release of the drug was shown both in normoxia (21% oxygen) and hypoxia (1% oxygen) conditions. Although these data are still preliminary, this pro-drug strategy appears promising as it compartmentalizes the action of a drug. This could be useful for inflammatory pathologies where NAMPT is overexpressed (**Figure 7**).

**Figure 8:**
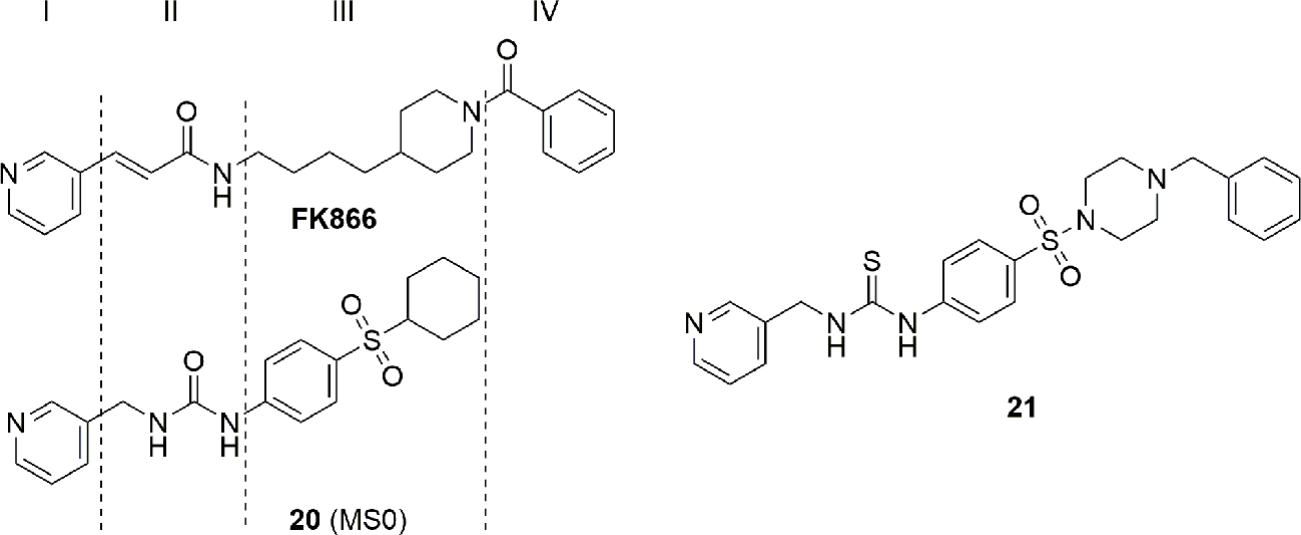
Hydrophobic interaction at the tail group of NAMPT inhibitors are essential for high inhibitory potency.

In a recent paper, Authors compared the X-ray binding pose of MS0 (**20**), a novel NAMPT inhibitor discovered by HTS, with FK866 in order to gain more insights on the molecular binding mode of NAMPT inhibitor for future optimizations. As shown in **Figure 8**, despite a very similar biological activity (IC_50_ = 9.08 nM for MS0 and IC_50_ = 1.60 nM for FK866), the length of the linker group is very different. If the lack of the hydrogen bond interaction of MS0 with Ser241 is easily rationalizable as thiourea is a poor hydrogen bond hydrogen acceptor compared to amides, the lack of the tail group prevents the formation of pivotal hydrophobic interactions which are useful both to increase affinity and to stabilize the inhibitor (Zhang S. et al., 2018). To note that following lead optimizations of MS0 aimed at increasing the hydrophobic binding interactions on the tail portion of the inhibitor, a more potent inhibitor **21** with an IC_50_ of 0.93 nM on NAMPT has been described (Xu et al., 2015) (**Figure 8**).

**Figure 9:**
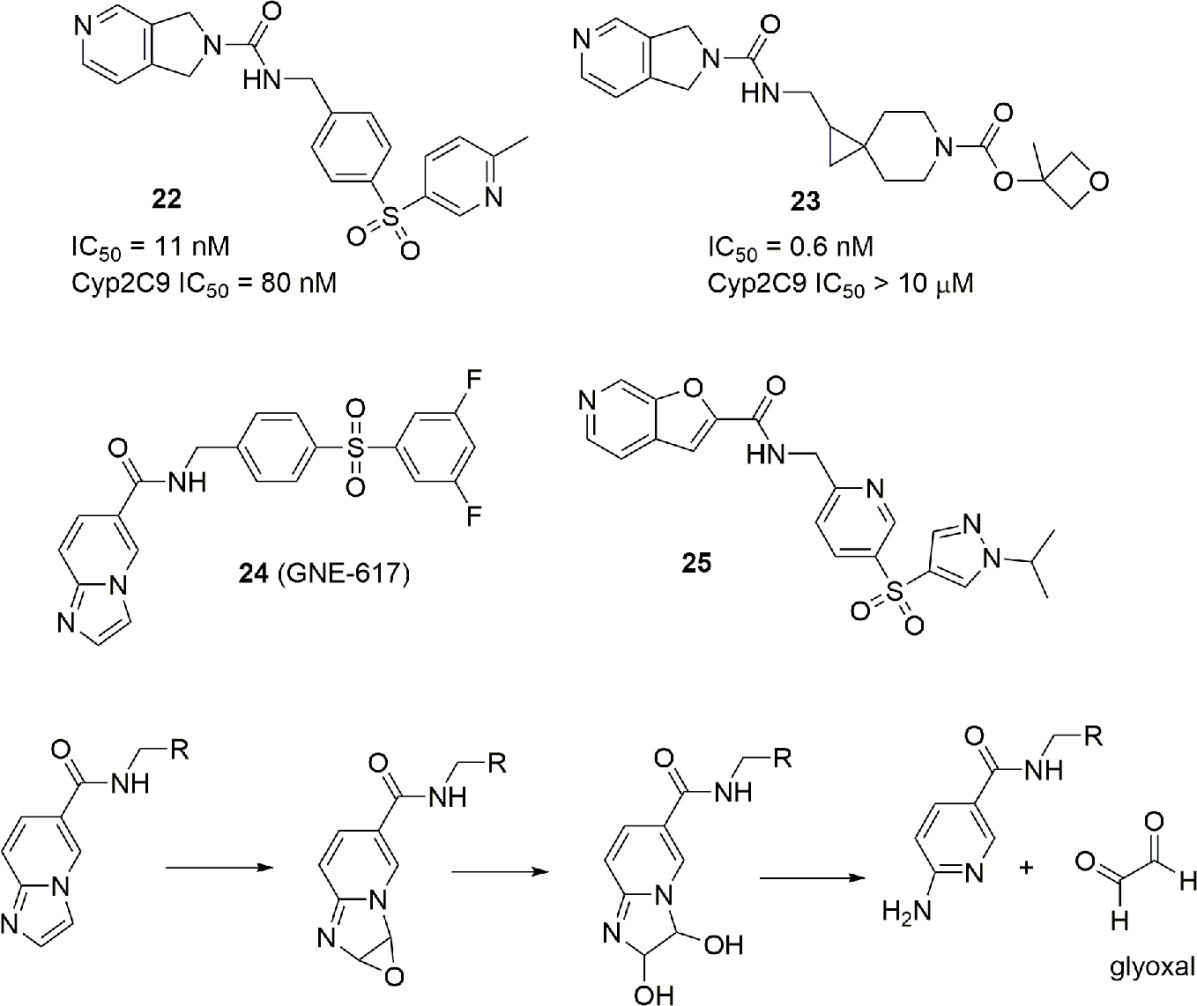
ADME optimization on potent NAMPT inhibitors.

The presence of a pyridine ring might be problematic due to the well-known ability of the nitrogen atom of pyridine to chelate the iron (II) of heme in several cytochromes. In particular, some NAMPT inhibitors such as **22** have shown to be potent inhibitors of CYP2C9. Although, for anticancer drugs, lack of inhibition of cytochromes involved in metabolism is not a mandatory requirement to advance to clinic, an industrial group reported how the modification of the linker and the tail group can suppress the inhibitory activity of the pyridine cap group on CYP2C9 retaining the potency as a NAMPT inhibitor (compound **23**) (Zak et al., 2016) (**Figure 9**).

NAMPT inhibitors are characterized not only by cytochrome inhibition but also by scarse aqueous solubility and formation of toxic metabolites. For example, imidazopyridines such as GNE-617 (**24**) can suffer of toxic metabolism, in particular with the formation of reactive glyoxal (**Figure 9**). For this reason, extra SAR studies have been presented on the potent and previously disclosed NAMPT inhibitor GNE-617 (**24**). Furthermore, in order to improve aqueous solubility, a strategy to reduce the number of aromatic rings, and consequently, the logD has been used. This effort brought to the identification of molecule **25**, which is characterized by a good water solubility and no toxic formation of metabolites (Zak et al., 2015) (**Figure 9**).

**Figure 10:**
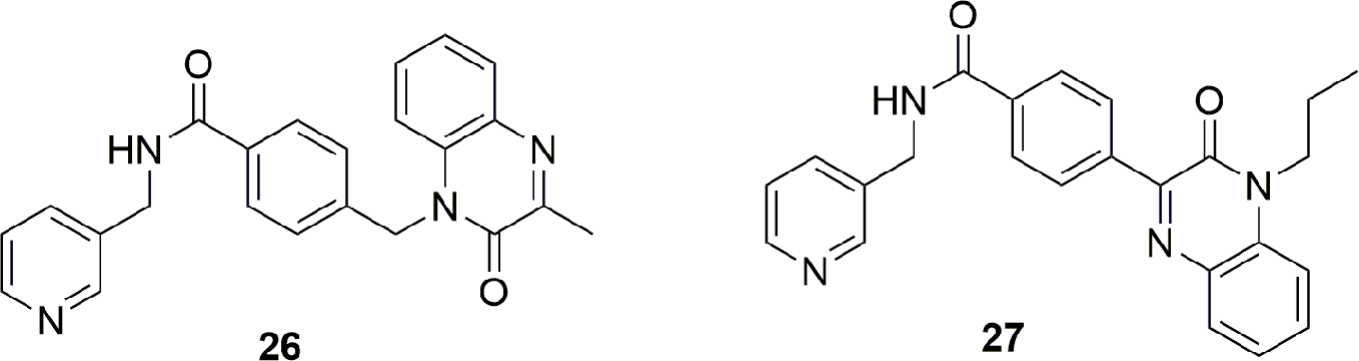
Compound **27** is a potent and fluorescent NAMPT inhibitor.

Last, from a screening of 3000 molecules as potential NAMPT inhibitors, two molecules (**26** and **27**) emerged with an *in vitro* inhibitory activity between 100 and 200 nM. Most importantly, the molecule named M049-0244 (**27**) was fluorescent and might therefore be useful in studies of localization of NAMPT inside and outside cells. Although other fluorescent probes linked to NAMPT inhibitors were already synthesized, the advantage with this class of inhibitors was that the fluorescent portion is also the tail group of the molecule, and this makes these compounds more drug-like compared to other reported probes (Wang et al., 2015) (**Figure 10**).

#### Molecules Which Contravene the Classical Pharmacophore Model

Crystallographic data of FK866 bound to NAMPT indicate that the pyridine ring is in a π stacking position between Tyr18 and Phe193, mimicking the position of nicotinamide. Furthermore, the pyridine nitrogen atom is involved in a hydrogen bond with the phenol of Tyr18. These data, along with the pyridine or pyridine-like NAMPT inhibitors discovered to date, seem to corroborate the idea that a pyridine ring as cap group is mandatory for NAMPT inhibition. To note, that the nitrogen atom can also been phosphoribosylated once bound to the enzyme, trapping the molecule inside the cell and boosting its biological action (Oh et al., 2014). This phosphoribosylation was thought indispensable to generate potent inhibitors (Sampath et al., 2015).

**Figure 11:**
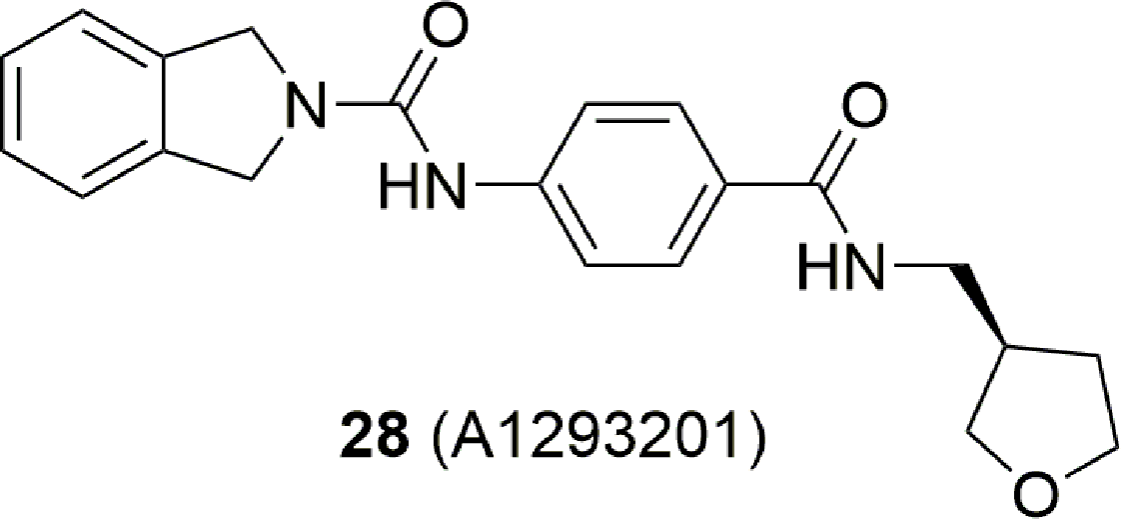
Pyridine cap group is not necessary to obtain potent NAMPT inhibitors.

Over the last years, this dogma was undermined by the identification of potent NAMPT inhibitors which did not contain a pyridine ring. For example, A-1293201 (**28**) (**Figure 11**) is cytotoxic and able to deplete the NAD levels (Guo et al., 2017; Wilsbacher et al., 2017).

This work has two merits: (i) it shows that the phosphoribosylation on the nitrogen atom of pyridine is not mandatory to obtain *in vivo* cytotoxic NAMPT inhibitors as previously postulated (Oh et al., 2014); (ii) the lack of the pyridine cap group yields molecules able to overcome the Y18C point mutation. Indeed, as previously discussed in this review, the nitrogen atom of pyridine acts as a hydrogen bonding acceptor with Tyr18, forming stronger π-stacking interactions with Tyr18 compared to a phenyl ring. In the Y18C point mutation, no π stacking interactions are possible as tyrosine has been replaced with a cysteine with negative consequences on the binding affinity for those inhibitors for which the affinity mostly depends on π stacking interaction. To note that the crystallographic pose between A-1293201 and the wild type enzyme shows how the isoindoline ring occupies the nicotinamide binding site, and it is parallel to Tyr18 participating in a weak π stacking interaction counterbalanced by an extra hydrogen bond between the enzyme and the distal secondary amide.

**Figure 12:**
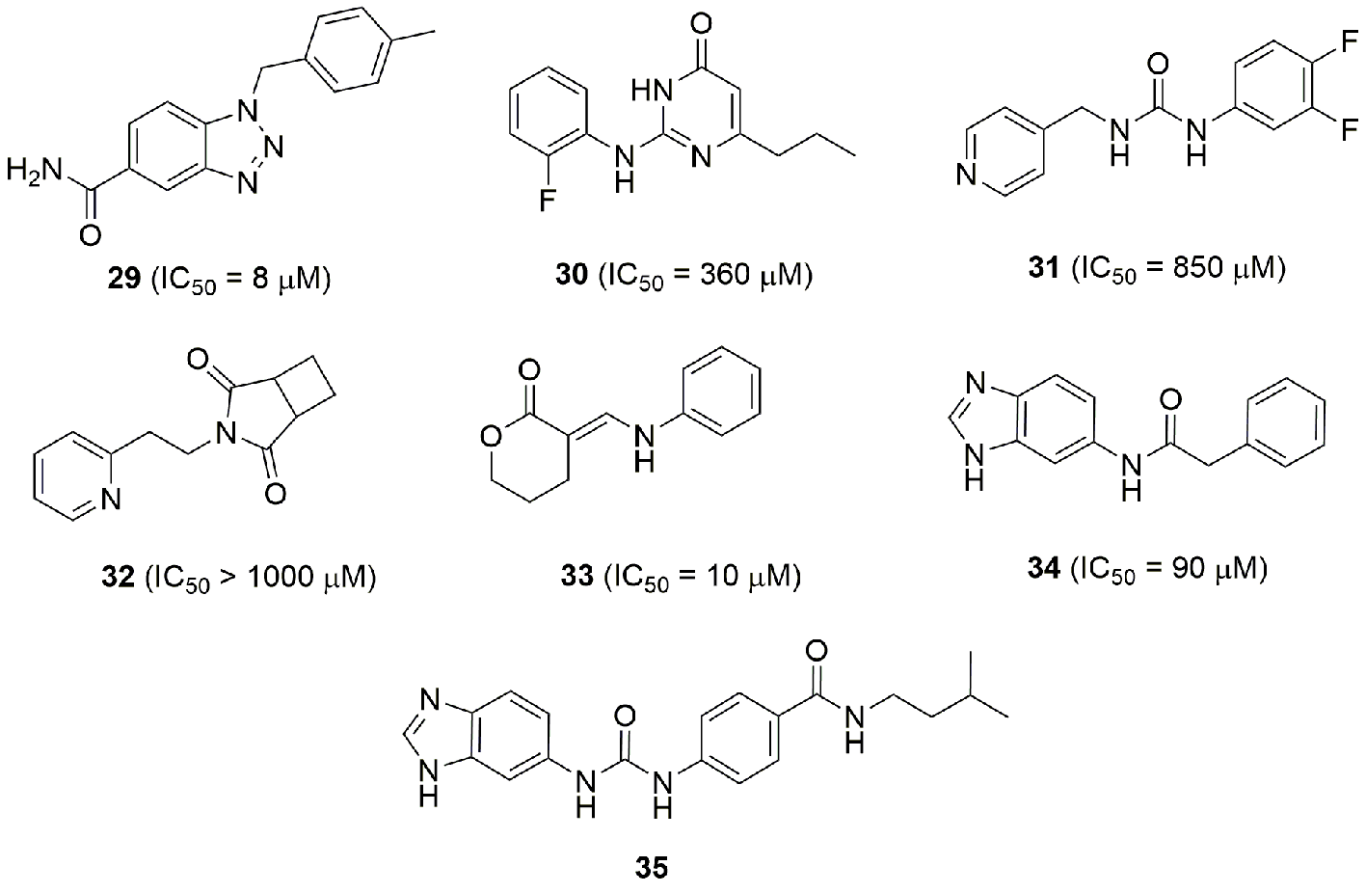
Fragment and structure base strategies allowed the identification of potent NAMPT inhibitors.

Combining the fragment-based and structure base strategy other NAMPT inhibitors have also been identified (Korepanova et al., 2018). In brief, six fragment binders (**29**–**34**) (**Figure 12**) with an activity between 8 and 1000 µM were identified using NMR and TR-FRET techniques. Two of these were then successfully crystalized with NAMPT, while the other four were docked. The comparison of their binding poses with those of FK866, led then to the design of compound **35** which has IC_50_ of 80 nM on the enzyme (**Figure 12**). It is interesting to note that apart molecules **31** and **32** which possess a pyridine cap group, the others present a different cap group. The crystallographic poses of **30** and **33** indicate the possibility that the binding pocket can accommodate a phenyl ring at the same position of pyridine. The most potent identified compound bears a benzoimidazole cap group and docking studies demonstrated that the nitrogen atom of this heterocycle nicely overlays with the nitrogen atom of pyridine.

**Figure 13:**
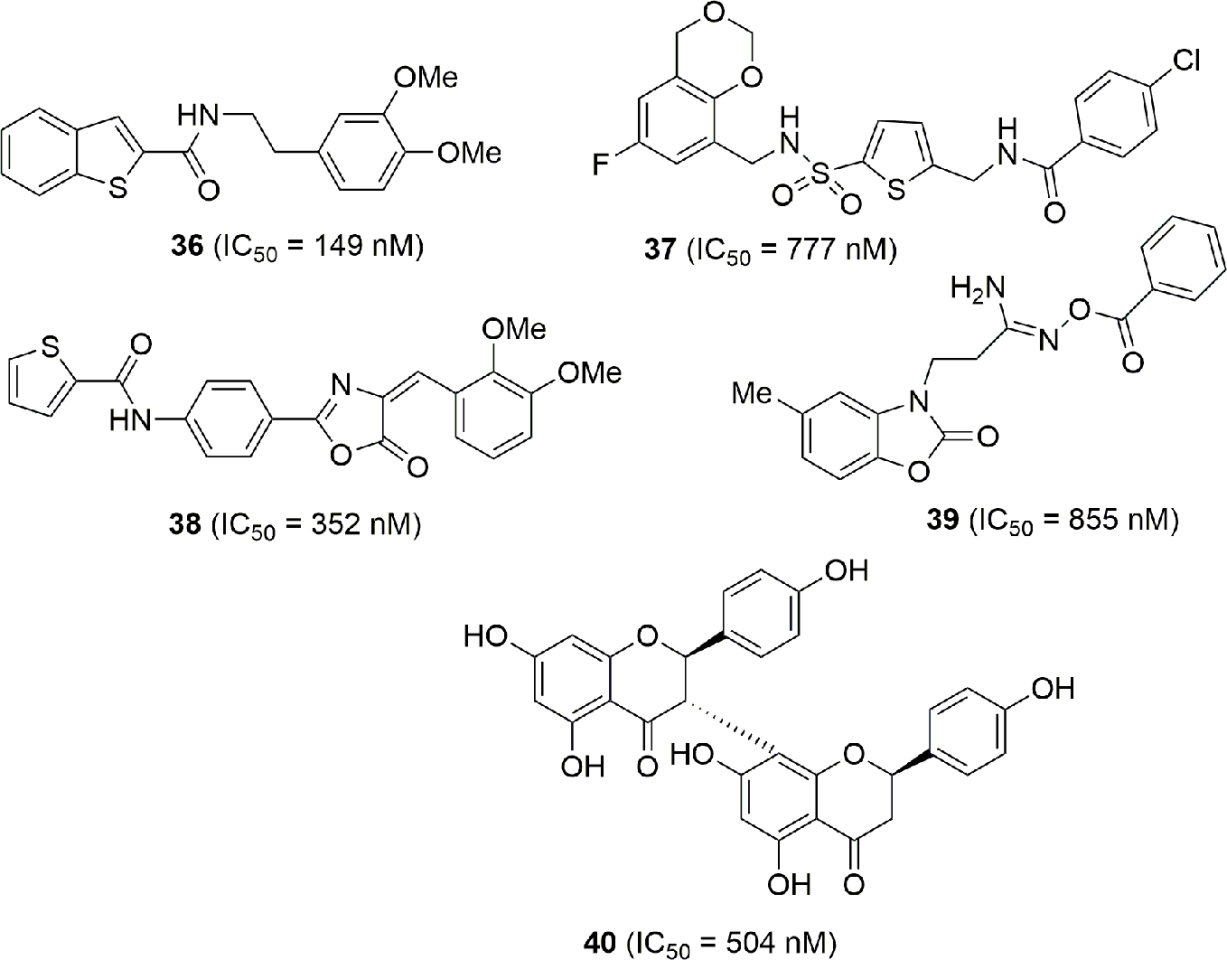
NAMPT inhibitors discovered *via* HTS.

In the high throughput campaign that led to the identification of MS0 (**20**), that falls in the classical NAMPT inhibitor structure, other molecules endowed with NAMPT inhibitory activity not correlated with classical NAMPT inhibitors were identified (**36**–**40**). Although the lack of the pyridine cap group reduces the efficiency, they still maintain potency and might be considered as novel lead compounds for optimization (Xu et al., 2015) (**Figure 13**).

**Figure 14:**
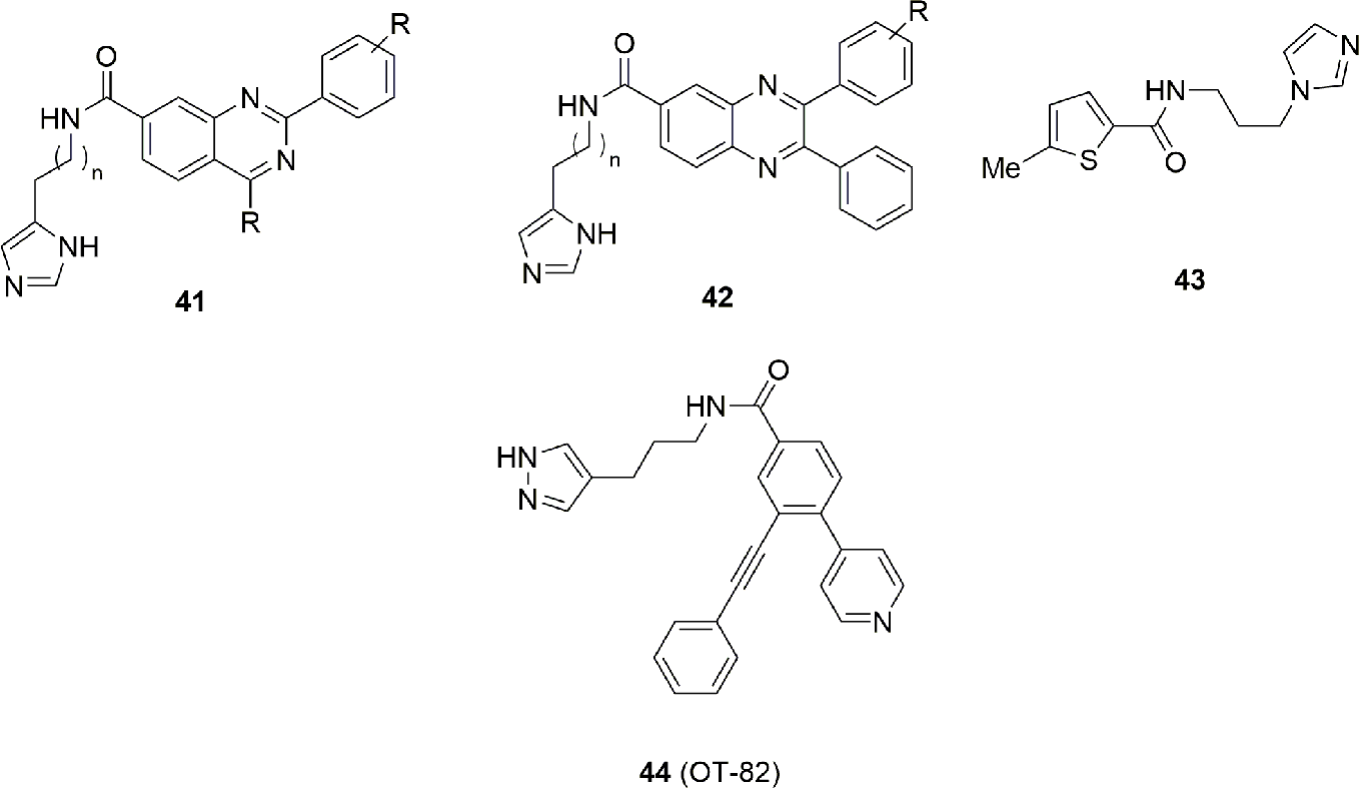
Imidazole/pyrazole group is a pharmacophoric element mandatory for this class of NAMPT inhibitors.

Imidazole has been used as a bioisosteric replacement of pyridine in two patents (Gigstad et al., 2015; Freeze et al., 2016), represented with the structures **41** and **42** in **Figure 14**. While no direct inhibitory data are available for each compound, the authors stated that many of them possess an IC_50_ under 10 nM.

Lack of structural information makes it difficult to speculate whether the imidazole behaves as a cap or a tail group. Chemical manipulation of **36** brought to the identification of compound **43** with an IC_50_ = 170 nM and an *in vitro* antitumor activity of 4 µM on HepG2 cancer cell line (Chen et al., 2016). Binding studies suggest that both **36** and **43** have a similar binding mode with FK866. In particular the (benzo)thiophene group intercalates between Phe193 and Tyr18, while the amide group is involved in an hydrogen bonding interaction with Ser275. Imidazole is directed toward the solvent exposed region being furthermore involved with a hydrogen bond with His191 (**Figure 14**).

Very recently Korotchkina et al. (2020) conducted a systematic search for novel agents selectively toxic to cells of hematopoietic origin. Chemical library screenings followed by hit-to-lead optimization identified OT-82 (**44**) (**Figure 14**), a small molecule characterized by a pyrazole group. The spectrum of OT-82 cytotoxicity was determined *in vitro* toward human cell lines derived from 12 hematological malignancies and 17 non-hematological malignancies cancers.

The average IC_50_ for OT-82 was significantly higher in non-hematological malignancies cancer cells compared with hematological malignancies cancer cells (13.03 ± 2.94 nM vs 2.89 ± 0.47 nM).

In toxicological studies conducted in mice and non-human primates, OT-82 showed no cardiac, neurological or retinal toxicities observed with other NAMPT inhibitors. Hematopoietic and lymphoid organs were identified as the primary targets for dose limiting toxicity of OT-82 in both species. These results reveal strong dependence of neoplastic cells of hematopoietic origin on NAMPT and introduce OT-82 as a promising candidate for the treatment of hematological malignancies (Korotchkina et al., 2020). A new clinical trial (NCT03921879) is now recruiting patients for OT-82 evaluation for relapsed and refractory lymphoma (**Table 1**).

#### Dual Inhibitors

There is a strong rationale to believe that NAMPT inhibitors might render better if in conjunction with other drugs, reducing the metabolic ability, and therefore the defense potential of cancer cells. For example, the combination between a NAMPT and a PARP inhibitor has been shown to synergize increasing the DNA damage and apoptosis of cells (Heske et al., 2017). The possibility to capitalize on a tool that weakens cells by reducing its metabolism in combination with agents that present a different mechanism of action has been investigated by numerous Authors, as depicted in **Table 2**.

**Table 2:** Evidences that NAMPT inhibitors may synergize with a number of other agents when used in combination.

Therapeutic Agent	Mechanism	Cell Lines	Therapeutical effects	Reference
Olaparib	PARP inhibitor	CAL51; HS578T; MDA-MB-231; MDA-MB-468; SUM149; MDA-MB-436	Sensibilization to olaparib in TNBC	(Bajrami et al., 2012)
AraC daunorubicin 1-methyl-3-nitro-1-nitrosoguanidinium (MNNG) melphalan	Antimetabolite; DNA alkylating agent; PARP activating agent;	THP-1 K562	Accelerations of cell death with AraC e daunorubicin and potentiation of DNA repair with MNNG	(Pogrebniak et al., 2006)
Verapamil	ABCB1-transporter inhibitor	HCT116	Reduced resistance to FK866	(Ogino et al., 2018)
Etoposide cisplatin	Topoisomerase inhibitor; DNA-alkylating agent	SH-SY5Y	FK866 potentiates DNA damage of etoposide and cisplatin accelerates NAD depletion	(Travelli et al., 2011)
Anti-PD1	Checkpoint inhibitor	MN/MCA1 in NAMPTf/f and NAMPTf/fLysMCre+/− mice	Enhanced anti-tumor efficacy, reduced metastasis	(Travelli et al., 2019b)
TRAIL	Tumor necrosis factor-related apoptosis-inducing ligand	Jurkat; PEER; H9; MOLT4; Namalwa	Increased autophagy	(Zoppoli et al., 2010)
EX527, sirtinol, cambinol, vorinostat, valproic acid, and butyrate	HDAC and sirtuin inhibitors	Primary AML cells; Jurkat 697; U937	Antileukemic effect	(Cea et al., 2011)
JPH203 l-asparaginase	LDHA inhibitors	CCRF-CEM MDA-MB-231	Reduced glycolysis and lactate production acquired with resistance to FK866	(Thongon et al., 2018)
Bortezomib	Proteasome inhibitor	MM.1S MM1R	Reduction of bortezomib resistance	(Cagnetta et al., 2013)
Rituximab	Anti-CD20	Burkit lymphoma Diffuse large B-cell lymphoma	Increase autophagy, caspase-3 activation, mitochondrial depolarization, and ROS production	(Nahimana et al., 2014)
Fractionate radiation	Radiation	PC3 LnCap	NAD depletion enhances radiation response	(Zerp et al., 2014)
Cyclosporin-A verapamil PGP-4008	Pgp inhibitors	OCI/AML2, OCI/AML3, HL-60, HEL, KG1a, SET1, MV4-11, MEC.1, MEC.2, LAMA-84, RPMI-8226, Dox40, Daudi, U937, Raji, SU-DHL1	Increased anti-tumor effect of APO866 decreasing resistance	(Cagnetta et al., 2015)
β-lapachone	Bioactivated by NAD(P)H:quinone oxidoreductase 1 (NQO1)	PDA cells; A549	Increased cell death	(Moore et al., 2015; Liu et al., 2016)
β-methylene adenosine 5′-diphosphate, APCP	CD73 inhibitor	OVCAR-3 cells	Marked potentiation of FK866 anticancer effects	(Sociali et al., 2016)
Gemcitabine	Antimetabolite	PDAC-derived PCCs	Potentiation	(Barraud et al., 2016)
Lu-DOTATATE	Radiolabeled somatostatin analogues	GOT1	Radiosensitivization	(Elf et al., 2017)
5-FU	Antimetabolite	MKN45, SGC7901, and BGC823	Suppressed cell migration and anchorage-independent growth	(Bi et al., 2011)
Pemetrexed	Antifolate	A549 H1299	PARP-1 activation and anti-tumoral effect	(Chan et al., 2014)
Temozolomide	Alkylating agent	U-251; T98 Patient-derived glioma line HT1080 (IDH1R132C)	Increased TMZ-induced apoptosis and necrosis.	(Feng et al., 2016; Tateishi et al., 2017)

The list has been extracted via searches from PubMed and may not be complete.

Given the efficacy of drug combinations, a strategy that has been pursued in drug development, also to overcome the pharmacokinetic problems linked to the simultaneous use of two drugs, was the synthesis of hybrid molecules able to display two different well-balanced mechanisms of action. The structure of all NAMPT inhibitors discovered to date allows for this type of med chem strategy. Indeed, if the pyridine ring is usually important as cap group to give potent compounds, the chemical nature of the so-called tail group which protrudes outside the enzyme is less mandatory as shown above and therefore amenable to recognize other targets.

STF-31 (**45**) (Kraus et al., 2018), an intrinsically dual acting drug with an inhibitory activity on NAMPT and GLUT1, and KPT-9274 (or ATG-019) (**46**) can be considered the archetypical examples of this novel class of compounds. The latter is a dual inhibitor of p21-activated kinase (PAK4) and NAMPT with potent cytotoxic activity on B-ALL cells and with the ability to function *in vivo* in a xenograft murine model. Several studies are emerging from literature, showing the impressive performance of this compound against a series of solid tumors (Abu Aboud et al., 2016). Importantly, KPT-9274 represents the first NAMPT inhibitor of the second wave to have entered clinical trials, although no data has so far been presented (**Table 1**). In the Phase I study, the drug is used as a single agent in the presence or absence of nicotinic acid (https://clinicaltrials.gov/ct2/show/NCT02702492).

**Figure 15:**
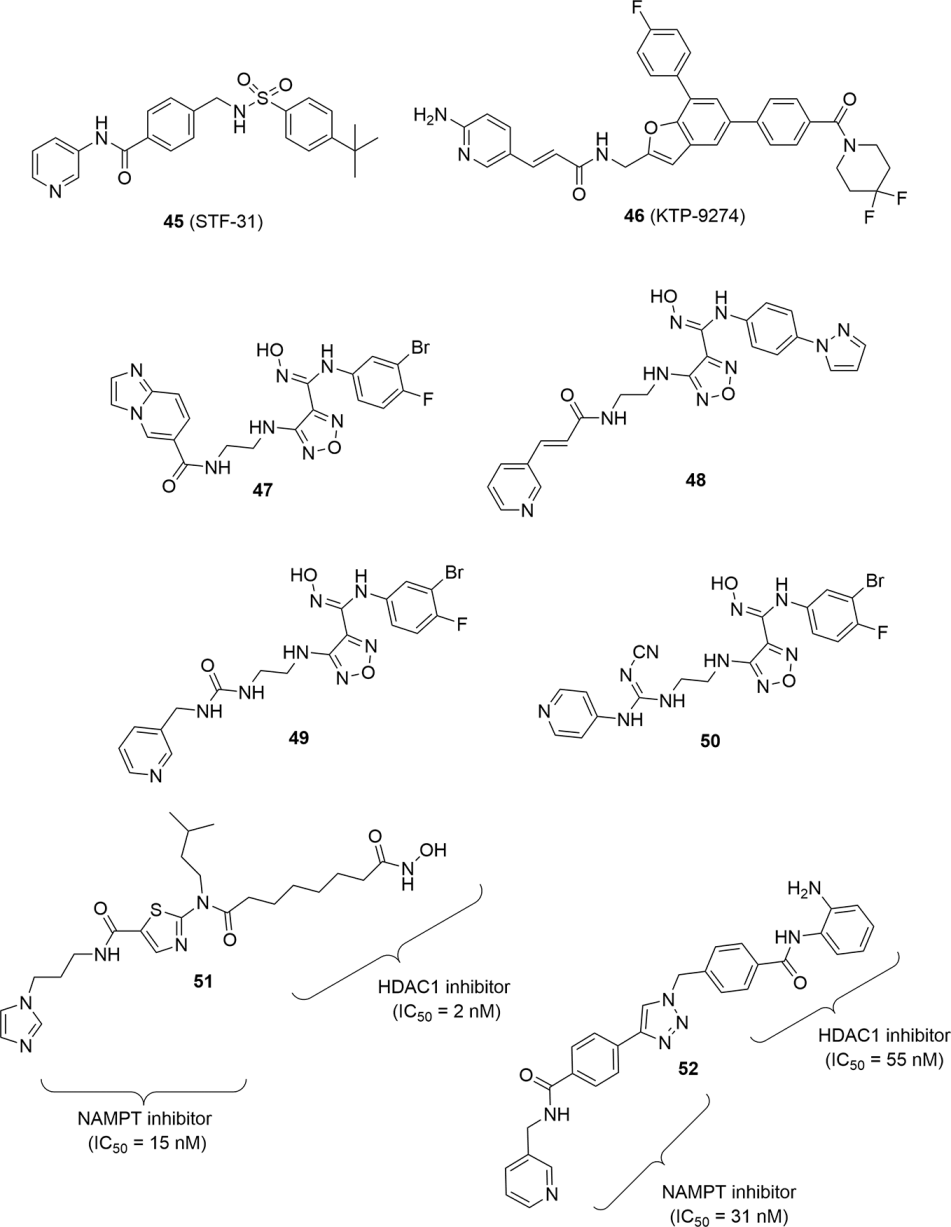
PAK4-NAMPT hybrid inhibitor, IDO1-NAMPT hybrid inhibitors and HDAC-NAMPT hybrid inhibitors.

Apart from this compound, to date this strategy has been used for the construction of hybrid molecules with NAMPT and IDO-1 inhibitory activity or NAMPT and HDAC inhibitory activity. For example, pharmacophoric group of the IDO-1 inhibitor epacadostat has been joined with pyridine derivatives as cap groups (Jiang et al., 2018). These hybrids (for example, **47**–**50**; **Figure 15**) showed stronger anti-tumor activity by inhibiting the biosynthesis of NAD^+^, exhibiting stronger tumor-suppressing activity and promoting T cell proliferation by inhibiting IDO activity. It should be nonetheless noted that T cell proliferation has been shown to be also an indirect property of pure NAMPT inhibitors *in vivo via* the inhibition of MDSC activity (Travelli et al., 2019b).

Finally, two different NAMPT-HDAC hybrid inhibitors have been disclosed by the same research group again using a pharmacophore fusion approach. The rationale for this fusion was the observation that NAMPT inhibitors like FK866 were able to enhance the inhibitory effect of HDACs being synergic in the anticancer effect. In particular compounds **51** and **52** proved to be very potent and well-balanced hybrids (**Figure 15**). Both compounds were evaluated *in vivo* for their antitumor efficacy in HCT116 tumor xenografts in nude mice showing a remarkable reduction of tumor growth superior with respect to SAHA or FK866 when administered as single therapy. To note that the hybrid molecule **52** was easily assembled using click chemistry as linker group (Dong et al., 2017; Chen et al., 2018).

### Strategies to Target the Extracellular Form of NAMPT

As mentioned previously, all efforts to develop novel inhibitors fall short of understanding the role that these inhibitors have on the activity of the extracellular form, which might be a target in its own right.

Nonetheless, sole targeting of the extracellular protein has now been attempted *via* the development of neutralizing antibodies. Garcia (Camp et al., 2015) first introduced this possibility in a model of ventilator-induced inflammatory lung injury, in which, the antibody reduced inflammation in mice and decreased NF-κB phosphorylation in human lung endothelial cells. In parallel, we started to develop a novel monoclonal antibody (C269) that neutralizes *in vitro* the cytokine-like action of eNAMPT and that reduces its serum levels in rodents. Of note, this newly generated antibody is able to significantly reduce acute and chronic colitis in both DNBS- and dextran sodium sulphate- (DSS) induced colitis (Colombo et al., 2020), demonstrating the eNAMPT participation in inflammatory bowel disease (IBD) and the therapeutic potential of its neutralization in this pathology.

While no data are at present available in cancer, there are ample evidences that the extracellular form is involved (as described above and in (Grolla et al., 2015; Dalamaga et al., 2018; Travelli et al., 2018; Audrito et al., 2019; Ji et al., 2019); and it would be a worthwhile effort to verify its neutralization in this field.

The low levels of PRPP in the extracellular space make it difficult to hypothesize that the enzymatic activity of NAMPT is necessary to extrinsicate its cytokine role, although this might not be true in the tumor microenvironment (because of the presence of necrosis areas) or in vesicles. Recently, indeed, Yoshida demonstrated that eNAMPT is contained exclusively in extracellular vesicles (EVs) in mice and humans and that supplementing eNAMPT in EVs improves physical activity and extends lifespan in mice (Yoshida et al., 2019).

Yet, whether the enzymatic activity is required in the extracellular space has never been formally tested with drugs, due to the difficulty of dissecting the effect of the known NAMPT inhibitors, that freely diffuse across the membrane. Furthermore, also in the plausible case that the enzymatic activity is disposable, it has never been ascertained whether NAMPT-inhibitor complexes still retain their cytokine-like role, as it is possible that the conformational changes may modify this. For this reason, Travelli et al. (2019a) first described extracellular inhibitors, incapable of crossing cell membranes due to their polarity (**4**; **Figure 4**). While these compounds would be intriguing to investigate the effect of eNAMPT, this has so far not been done. Preliminary unpublished data from our lab has shown a significant toxicity of compound **4**, that may be reconducted to an unknown off-target effect (although this was not observed with cell-permeable compounds) or might disclose the importance of extracellular NAMPT (CT and GC, personal communication).

## Conclusion

The initial hype on NAMPT inhibitors brought to several compounds entering clinical trials. It is possible that such enthusiasm was premature, and more efforts at the time should have been devoted to better understanding the biology of NAMPT. Indeed, it is now emerging that it is much more than a simple workhorse to replenish NAD. The complexity of this enzyme is exemplified by the fact that, while the role as an extracellular cytokine is yet not fully elucidated, it has just emerged that it can also shuttle to the nucleus in cells to provide NMN/NAD on site (Svoboda et al., 2019; Grolla et al., 2020). Although more information has to be gathered, it is now apparent that the hypothesis of using NAMPT inhibitors as a single agent is possibly flawed by the occurrence of side effects. Better, therefore, is the possibility to use it as a combination therapy in which tumors are NAMPT-dependent and in which MDSCs play a role in immune escape. Further biological elements can also be determined: for example, if NAMPT is fundamental for nuclear NAD, then it could be envisaged that PARP inhibitors could be good therapeutic partners (Bajrami et al., 2012; Heske et al., 2017). This is just a demonstration on how understanding the biology of the protein can increase significantly the efficacy of medicinal chemistry programs. The use of the drug in combination would possibly also reduce toxicity, although it is at present unclear if the immunotherapeutic effect is achieved at similar doses in humans or if dose adjustments are necessary. The choice of dual inhibitors, therefore, appears to go in this direction. Lastly, the possibility to target only the extracellular form of NAMPT, either with antibodies or small chemical entities is intriguing. The last element that should be considered is NAPRT, that is now emerging as a new possible target and for which no specific inhibitors are at present available.

In conclusion, the time is mature to develop and define place in therapies for new NAMPT inhibitors, but their development and characterization should not solely be based on cytotoxicity of tumoral cells or on xenograft models, but should take into account all the lessons learnt so far. This, we hope, will create a new wave of molecules entering clinical trials.

## Author Contributions

AmG, UG, and GT proposed, collected information, and wrote the manuscript. GC and CT collected and analyzed the information. ArG supervised the conception and writing of the manuscript.

## Funding

The research was supported by an AIRC grant to ArG (AIRC IG2018 21842), by a PRIN grant from the Italian Ministry of Health to ArG (PRIN 2017 CBNCYT).

## Conflict of Interest

The authors declare that the research was conducted in the absence of any commercial or financial relationships that could be construed as a potential conflict of interest.
